# Effects of Different Training Intensity Distribution in Recreational Runners

**DOI:** 10.3389/fspor.2019.00070

**Published:** 2020-01-15

**Authors:** Luca Festa, Cantor Tarperi, Kristina Skroce, Antonio La Torre, Federico Schena

**Affiliations:** ^1^Department of Neuroscience, Biomedicine and Movement Sciences, University of Verona, Verona, Italy; ^2^Department of Clinical and Biological Sciences, University of Turin, Turin, Italy; ^3^Department of Biomedical Sciences for Health, University of Milan, Milan, Italy

**Keywords:** intensity distribution, focused training, polarized training, running economy, recreational runners

## Abstract

**Purpose:** To compare the impact of two different training intensity distributions in terms of conditional and performance parameters and spent time to training in recreational athletes.

**Methods:** Two different training intensity distribution model were performed for 8 weeks by 38 recreational runners. Runners recruited were randomly assigned to 2 different training models based on HR intensity detected with maximal test. The percentage distribution splitted in zone 1, 2, and 3 were by 77/3/20 and 40/50/10 in polarized endurance training group (PET) and focused endurance training (FOC) group, respectively. Programs were balanced for total training impulse (TRIMP). To evaluate effects of training, before and after treatment were performed a maximal exercise test to determine Maximal Oxygen Uptake (V'O_2max_), Ventilatory Threshold (VT), respiratory-compensation point (RCT) Running Economy (RE), and 2 Km performance. To investigate the effects of training on muscular performance were performed one repetition maximum (1 RM), squat jump (SJ), and counter movement jump (CMJ).

**Results:** Both groups significantly improved their velocity at V'O_2max_ (3.2 and 4.0%), at VT (4.0 and 3.2%), RCT (5.7 and 3.4%), the average velocity in 2 Km performance (3.5 and 3.0%) and RE (−5.3 and −8.7%) for PET and FOC, respectively for each variable. No differences were found between the groups on any parameter investigated except about the total training time (PET = 29.9 ± 3.1 h and FOC = 24.8 ± 2.0 h).

**Conclusion:** Focused Endurance Training obtains similar improvements than Polarized Endurance Training saving 17% of training time in recreational runners.

## Introduction

To maximize endurance performance, coaches, and scientists can manipulate the characteristics of training: intensity, duration, and frequency of training session during the entire training process (Seiler, 2010). There is general agreement on the physiological factors limiting performance (di Prampero, 2003; Coyle, 2007), however there is still no agreement on how the daily training process must be organize to improve physiological factors and performance.

In recent years many studies have suggested that training intensity distribution plays a key role on endurance training adaptation not only in elite (Seiler and Kjerland, 2006; Seiler et al., 2007; Laursen, 2010; Seiler, 2010; Ingham et al., 2012), but also in well-trained recreational athlete (Esteve-Lanao et al., 2005, 2007; Neal et al., 2013; Muñoz et al., 2014; Stöggl and Sperlich, 2014). Training intensity distribution (TID) in endurance training programs is determined from the percentage of time spent exercising at low (zone 1, typically identified below the lactate threshold (LT), or ventilatory threshold (VT); moderate (zone 2, typically located between LT and maximal lactate steady state (MLSS) or respiratory-compensation threshold (RCT); and high (zone 3, typically above MLSS or RCT) intensities (Seiler and Kjerland, 2006; Faude et al., 2009). Although the topic is widely debated, just few studies at the moment are referred to running, while the reference studies for TID refer to other endurance sports such as cross country ski and cycling. Scientific literature has identified two well-differenced training models based on intensity distribution (Esteve-Lanao et al., 2007; Neal et al., 2013). First, a polarized training model (PET) that consist of a high percentage of exercise time at low exercise intensity (75–80%) and the remainder spent at high intensity (20–25%). In contrast, the second model is a traditional threshold training distribution (THR), in which the time distribution is: 45% at low, 35% moderate, and 20% high intensity, respectively. Several studies have observed the TID of well-trained and highly trained endurance athlete in different disciplines (Seiler and Kjerland, 2006; Esteve-Lanao et al., 2007; Plews and Laursen, 2017; Kenneally et al., 2018) and there are substantial evidences that PET may optimize adaptation to exercise while providing an acceptable level of training stress. Different studies have investigated the relation between adaptations and intensity of training and they affirm that LT is positive affected when a high proportion of training is conducted at low intensity (Esteve-Lanao et al., 2005, 2007; Ingham et al., 2008), they are suggesting that the proportion of time in zone 1 is a key aspect that drives endurance adaptations and performance outcomes. However, several other studies have observed improvements on 40 km time trial when high intensity training (zone 3) is added into the schedule of well-trained cyclist (Lindsay et al., 1996; Westgarth-Taylor et al., 1997; Weston et al., 1997). As Seiler states for highly trained athletes training 10–25 h/week, polarized intensity distribution may allow maximal adaptive signaling while minimizing autonomic and hormonal stress responses and reducing the risk of overtraining (Foster, 1998; Esteve-Lanao et al., 2007).

For recreational athletes is still unknown what intensity distribution is optimal or if the intensity distribution is or is not critical. In the study of Muñoz et al. (2014), polarized training model shown a better impact on 10 km performance in recreational runners compared with the threshold training model after 10 weeks of practice (−3.5% for THR and −5% for PET), but they conclude that there is not enough evidence in the overall findings to support one approach over the other.

To try to bring evidence in favor of the correct approach, the goal of the present study was to compare the conditional and performance effects of PET training model with a focused (FOC) training model on changes in limiting factors.

## Materials and Methods

### Experimental Approach to the Problem

A two groups pretest-posttest design was used. The effects of different training were verified on the performance in 2 km and through the analysis of changing in the value of limiting factors measured during the test in laboratory pre and after intervention. The main difference between training models is the time spent in zone 2. One group of athletes performed a relatively higher percentage of their total training volume in zone 1, below their VT. The second group trained 50% of total training volume in zone 2, between VT and RCT, while training less in zone 1 and zone 3. To compare training the total training load (intensity × volume) was balanced using a modified version of the training-impulse approach (TRIMP) (Foster et al., 2001).

### Subjects

Forty-three recreational runners were recruited to participate in this study. All subjects had been training consistently for >4 years (average experience 6.4 ± 1.6 years) and mean training volume in terms of duration before the study was 3.2 ± 0.5 h per week. Furthermore, they raced a half marathon a week before the beginning of the study. The University Ethical Committee approved the protocol (Prot. N. 165038, 28/06/2016) and the participants gave their written consent before taking part.

Runners recruited and randomly assigned to 2 different training groups (each *n* = 19), see Table 1, for an 8-week period. Dropout rate for the FOC group was 21% (two subjects was excluded from the analysis due to training program adherence <96%; two abandoned the experiment for personal reasons). Dropout rate for the PET group was 5% (one subject abandoned the study personal reason). Groups' characteristics are displayed in Table 1. Groups were similar regarding age, body mass, height, and V'O_2max_ (see Table 1).

**Table 1:** Physical characteristics of runners included in the analysis.

**Variables**	**Polarized endurance training (PET)**	**Focused endurance training (FOC)**
*N*. (m / f)	15/4	16/3
Age (yr)	43.2 ± 8.4	39.4 ± 8.5
Weight (kg)	72.0 ± 7.7	70.9 ± 10.1
Height (cm)	175.2 ± 5.9	172.5 ± 4.3
V'O_2max_ (ml min^−1^ kg^−1^)	52.9 ± 8.1	53.4 ± 8.3

### Training Plan and Prescription

**Figure 1:**
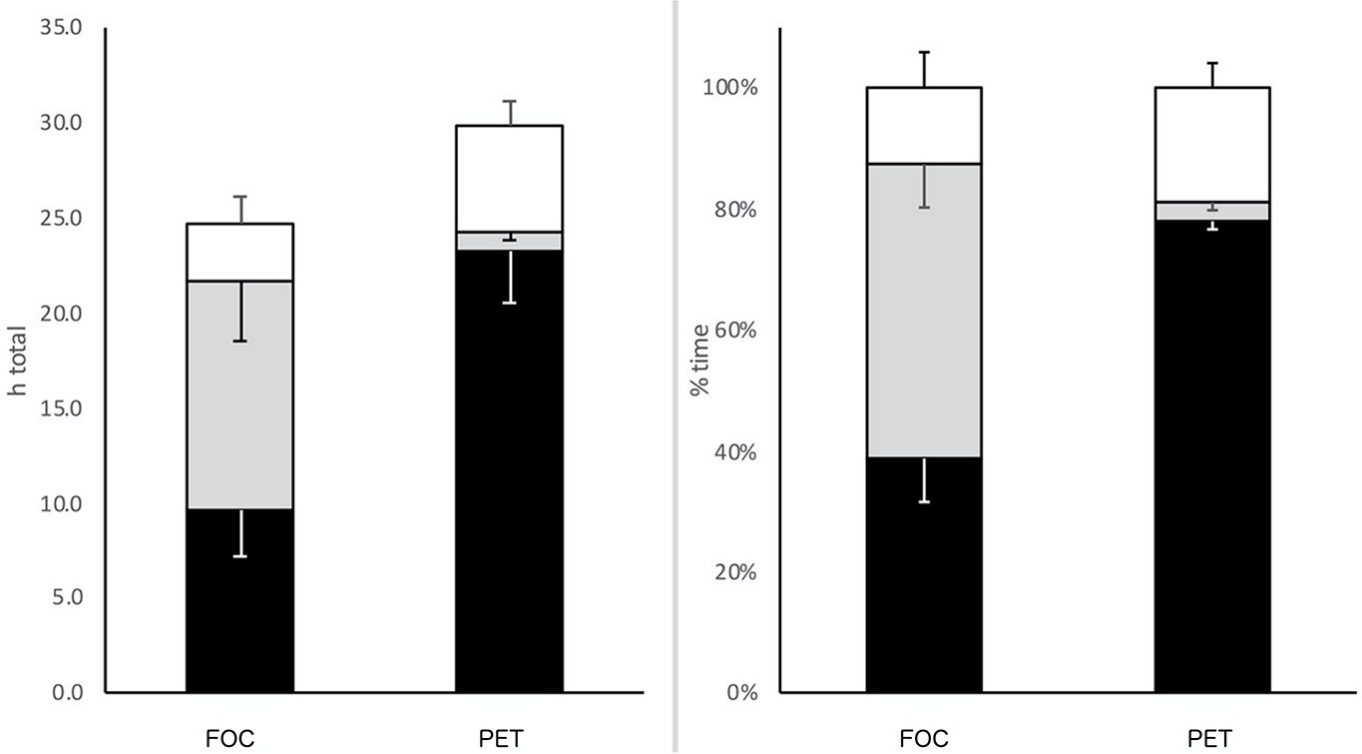
Total running time (h) on the left and percentage of TTT in each zone (%) on the right of different training groups.

The training plans were designed to reach a similar score for both total TRIMP accumulated over 8 weeks (2,492 ± 72 TRIMPs) and mean TRIMP accumulated each week (311 ± 9) (Table 2). We prescribed the training in terms of time goal rather than distance to track the relative time in each zone for each athlete and to control training load. The polarized endurance training (PET) was designed to achieve a total percentage distribution in zone 1, 2, and 3 corresponding to 77/3/20 based on HR at RCP. The second plan, focused endurance training (FOC), has a percentage distribution of 40/50/10 in zone 1, 2, and 3, respectively (Figure 1).

**Table 2:** Results of training load over the 8 week total training time (TTT).

	**Group**	
	**PET**	**FOC**
	**(*n* = 19)**	**(*n* = 19)**
Total running time	29.9 ± 3.1	24.8 ± 2.0
TTT in zone 1 (h)	23.3 ± 2.7	9.6 ± 1.2
TTT in zone 2 (h)	0.9 ± 0.4	12.1 ± 2.1
TTT in zone 3 (h)	5.7 ± 1.4	3.1 ± 1.4
TTT % in zone 1 (%)	78 ± 9.2	38.7 ± 9.6
TTT % in zone 2 (%)	3.1 ± 1.3	48.8 ± 12.8
TTT % in zone 3 (%)	18.9 ± 4.6	12.5 ± 5.7
Mean RPE session	60.9 ± 15.5	65.4 ± 14.6
Total TRIMPs (au)	2464.0 ± 124.0	2558.2 ± 10.9
Mean TRIMPs/wk (au)	308.0 ± 47.46	319.8 ± 28.1

The program was divided into 2 weeks of introductory period same for all and two 3 weeks microcycles following a 2:1 load structure. The relative intensity distribution of groups was maintained both in loading and unloading weeks.

The weekly schedule for PET group included four sessions, two of which hard with interval or repetition workout at high intensities, one at moderate average intensity and one easy run. For FOC group the training session was three and all the session have medium and long repetition at moderate intensity.

Moderate and high intensity is essential in a program since involve large muscle mass and could lead at a better resistance at fatigue during running performance (Boccia et al., 2017).

No strength training sessions were performed during the training period.

### Laboratory Testing

Laboratory, field running test and muscle function tests were performed during week 0 and week 9. The tests were separated by a 24-h resting period. All the tests were performed at the same time of the day ± 2 h in a climate-controlled laboratory (20–22°C, 55% humidity). The participants did not perform any physical activity in the 24-h resting periods and were requested to refrain from using caffeine containing food or beverages. All the subjects performed familiarization trials. The entire tests were randomized; however, the same order was respected in the pretest and posttest for everyone.

### Incremental Test to Exhaustion

The three intensity zones were establish based on the results of treadmill testing performed in laboratory at week 0. Maximal oxygen uptake (V'O_2max_) and heart rate (HR) was measured and recorded during a treadmill incremental maximal running test by breath by breath analysis of oxygen consumption and carbon dioxide production (Quark PFT; Cosmed, Rome, Italy). Before each test, flow meter was calibrated with a 3-L syringe, and the analyser was calibrated with known gas mixtures (16% O_2_ and 5% CO_2_) and environmental air (20.9% O_2_ and 0.03% CO_2_).

The protocol test was individualized for each subject to control the duration of each test. Therefore, the initial speed was determined by the subject capacity, and it was increased by 0.5 km h^−1^ every minute until exhaustion. The duration of the test was expected to be between 10 and 15 min. The treadmill (Run Race 800; Technogym, Gambettola, Italy) was maintained at 1% grade throughout the test, a standard method to simulate level running on treadmill. All subjects were familiarized to run on treadmill (Galbraith et al., 2014).

V'O_2max_ was defined as the highest 30 s average achieved during the test. The first ventilatory threshold (VT) was defined as an increase in V_E_ · V'O2-1 corresponding with a break in linearity in VE, but without increase in V_E_ · V'CO2-1. The respiratory-compensation threshold (RCT) was defined as the intensity where V_E_ · V'CO2-1 also began to rise. Two independent evaluators made the threshold determination. If the difference in VO_2_ values at VT_1_ and VT_2_ was higher than 200 ml min^−1^ between the 2 observers (% of agreement = 88%), a third was brought in Beaver et al. (1986).

### Running Economy

Running Economy (RE) was determined by measuring submaximal V'O_2_ during running on treadmill (Barnes and Kilding, 2015): 4 min at 1 km h^−1^ slower than the last individual marathon pace (8.9 ± 0.2 km h^−1^) after a standardized warm up (4′ at 90% of marathon pace). Before each test, flow meter was calibrated with a 3-L syringe, and the analyser was calibrated with known gas mixtures (16% O_2_ and 5% CO_2_) and environmental air (20.9% O_2_ and 0.03% CO_2_). During each test heart rate was monitored and recorded with Cosmed flow meter (Quark PFT, Cosmed, Rome, Italy). The RE was defined as the mean V'O_2_ collected at last 1 min of each running speed. The RE was measured at week 0 and after completing the training program at week 9. All tests were performed at the same time of the day for everyone.

### 2 km Performance

Before and after training after a standardized warm-up session the subjects participated in a 2 Km simulated race on 400 m running track. Performance time was the mean value between the time recorded by two people manually. The average difference time between the two evaluators was <0.3± 0.14 s. The race-test was conducted at the same day and hour of week.

### Estimation of One-Repetition Maximum

Maximal strength was estimated with a 6RM test on leg press machine. All subjects were positioned on a horizontal leg press (Technogym, Gambettola, Italy) and the knee angle (90°) was fixed to maintain the same position in all test occasions. After a 5-min warm-up (4 min moderate cycling + 1 min of free weight exercise) with and a correct rest period, the subject performed the first session with a preliminary load of 15 repetitions. Thereafter, the load was increased every step by 30% until the athlete could not successively complete a 6 RM repetition (American College of Sports Medicine, 2018). The 1 RM was estimated through a conversion table.

### Jumping Performance

All subjects performed a squat jump (SJ) and counter movement jump (CMJ) test. Vertical jump performance was assessed using the SJ and the CMJ test according to the procedures suggested by Bosco et al. (1983). Jumping height was calculated from flight time using kinematics equation (Lehance et al., 2005). Flight time was recorded using an infrared photocell connected to a digital computer (Optojump System, Microgate SARL, Bolzano, Italy). All tests were performed in a randomized order; however, the same order was respected during test after training period.

### Exercise Training Load

All subject recorded HR continuously during each training session over the training period. To assess the training load (intensity × training duration) for both, PET and FOC groups the TRIMP proposed by Foster et al. (2001) was used. This method was used by Muñoz et al. (2014) to estimate exercise load of 10 weeks of training in recreational runners (Muñoz et al., 2014) and also to monitor the exercise load of 3 weeks professional cycling race (Santalla et al., 2012). This method integrates heart rate data with volume and relative intensity to the three zones detected by the heart rate at VT and RCT. From the incremental test results, heart rate values for VT and RCT are determined and then quantified the time spent in each intensity zone: zone 1, HR below the VT; zone 2, HR between VT and RCT and zone 3, HR above RCT. TRIMP is computed by multiplying the accumulated time in each zone by an intensity weighted coefficient (1 for zone 1, 2 for zone 2 and 3 for zone 3) to obtain a score. Total TRIMP load is then obtained by summing the 3 zone scores.

### Internal Training Load Monitoring

During the training period, each session was recorded and uploaded on a network platform that allowed the recording of the time spent in each intensity zone during each session. A 100 point rating perceived exertion (RPE) (Borg and Kaijser, 2006) was obtained at the end of each session.

### Statistical Analyses

Data was presented as M ± SD. Assumptions verification were performed before each test. Normality distribution for each dependent variable were checked with Shapiro–Wilks tests. In the case of any normality violation, the non-parametric test version was applied. Independent samples *t*-tests were used to determine the significance of differences in the measured variables indicative of anthropometric and fitness levels before training between independent groups. To ensure that total training load and distribution in intensity zones were different were also compared total TRIMP and total time spent in zone 1, 2, and 3. A 2 × 2 mixed measure ANOVA was performed after training for all variable using Bonferroni's correction method. Differences between PRE vs. POST were reported in absolute values, the precision of estimates for absolute values was indicated with 90% confidence limits (CL), effect size (d), and benchmark for significance was set at *p* ≤ 0.05.

## Results

Normality was respected for each dependent variable (all *p* > 0.05).

Total training time over 8-weeks was significantly different and was 29.9 ± 3.1 h and 24.8 ± 2.00 for PET and FOC group, respectively. Weekly 308.0 ± 47.5 and 319.8 ± 28.1 and total 2464 ± 124.0 and 2558.2 ± 10.9 TRIMP scores were not different between the two groups (Effect size −0.65 *P* > 0.05). Total time spent in training zone 1 (PET = 23.3 ± 2.7 h vs. FOC = 9.1 ± 2.4 h, *P* < 0.0001), zone 2 (PET = 0.9 ± 0.4 h vs. FOC = 11.5 ± 3.2 h, *P* < 0.0001), and zone 3 (PET = 5.7 ± 1.4 h vs. FOC = 3.1 ± 1.4 h, *P* = 0.0001).

No significant difference was found in the comparison between the groups in any investigated variable before and after training. However, significant improvements from the pre-training to the post-training were observed in both PET and FOC in physiological parameters. For PET, there are significant improvements in speed at V'O_2max_ (vV'O_2max_) of 3.2% (12.9 ± 1.7 km h^−1^ vs. 14.3 ± 1.5 km h^−1^, *P* < 0.01), speed at VT of 4.0% (10.5 ± 1.2 km h^−1^ vs. 10.9 ± 1.2 km h^−1^, *P* < 0.001), speed at RCT of 5.7% (12.1 ± 1.5 km h^−1^ vs. 12.8 ± 1.4 km h^−1^, *P* < 0.01) RE of 5.3% (226.3 ± 35.2 vs. 214.3 ± 33.0 ml min^−1^ km^−1^, *P* = 0.04) and average velocity on 2 km performance 3.5% (13.8 ± 2.0 vs. 14.3 ± 1.7 km h^−1^). Also for FOC group were recorded significant improvements in the same variable at speed at vV'O_2max_ of 4.0% (13.8 ± 1.9 km h^−1^ vs. 14.3 ± 1.8 km h^−1^, *P* = 0.03), speed at VT_1_ of 3.2% (10.8 ± 1.4 km h^−1^ vs. 11.1 ± 1.5 km h^−1^, *P* = 0.04), speed at RCT of 3.4% (12.4 ± 1.7 km h^−1^ vs. 12.8 ± 1.7 km h^−1^) average velocity on 2 km performance 3.0% (13.9 ± 1.9 km h^−1^ vs. 14.3 ± 1.9 km h^−1^) (Tables 3, 4).

**Table 3:** Structural, functional, and performance results in the PET group.

	**Pretraining**	**Posttraining**	**Difference**	**Lower bound**	**Upper bound**	**Effect size (d)**
**STRUCTURAL**						
Weight (kg)	72.0 ± 7.7	71.8 ± 7.3	−0.22	−0.56	1.00	0.0
Fat mass (%)	19.9 ± 5.9	17.4 ± 4.9	−2.52	0.69	4.34	0.4^*^
**FUNCTIONAL**						
V'O_2max_ (ml min^−1^ kg^−1^)	53.0 ± 5.9	53.6 ± 4.8	0.63	−2.01	0.75	0.1
vV'O_2max_ (km h^−1^)	13.9 ± 1.7	14.3 ± 1.5	0.45	−0.67	−0.22	0.3^*^
vVT (km h^−1^)	10.5 ± 1.2	10.9 ± 1.2	0.42	−0.62	−0.22	0.3^*^
vRCT (km h^−1^)	12.1 ± 1.5	12.8 ± 1.4	0.68	−0.94	−0.43	0.4^*^
RE (ml kg^−1^ km^−1^)	226.3 ± 35.2	214.3 ± 33.0	−12.02	1.39	22.64	0.4^*^
1 RM leg press (kg)	223.7 ± 64.6	223.9 ± 61.1	0.23	−31.39	30.92	0.0
SJ (cm)	22.7 ± 4.6	23.3 ± 4.4	0.64	−2.03	0.75	0.1
CMJ (cm)	24.9 ± 5.3	24.9 ± 4.9	0.07	−1.41	1.26	0.0
**PERFORMANCE**						
Avg velocity 2 Km (km h^−1^)	13.8 ± 2.0	14.3 ± 1.7	0.48	−0.86	−0.11	0.1^*^

*Results are presented as mean ± SD*.

* *p value PRE vs. POST < 0.05*.

**Table 4:** Structural, functional, and performance results in the FOC group.

	**Pretraining**	**Posttraining**	**Difference**	**lower bound**	**Upper bound**	**Effect size (d)**
**STRUCTURAL**						
Weight (kg)	70.9 ± 2.5	69.9 ± 2.5	−1.0	0.08	1.87	0.1
Fat mass (%)	18.5 ± 1.8	16.9 ± 1.7	−1.6	−0.15	3.41	0.3^*^
**FUNCTIONAL**						
V'O2_max_ (ml min^−1^ kg^−1^)	53.7 ± 1.9	53.2 ± 1.9	−0.5	−1.00	2.00	0.1
vV'O2_max_ (km h^−1^)	13.8 ± 0.5	14.3 ± 0.4	0.5	−0.85	−0.15	0.3^*^
vVT (km h^−1^)	10.8 ± 0.3	11.1 ± 0.4	0.3	−0.65	−0.04	0.3^*^
vRCT (km h^−1^)	12.4 ± 0.4	12.8 ± 0.4	0.4	−0.67	−0.14	0.3^*^
RE (ml kg^−1^ km^−1^)	231.8 ± 9.1	211.6 ± 6.3	−20.2	8.88	31.54	0.6^*^
1 RM leg press (kg)	210.5 ± 18.8	193.8 ± 15.5	−16.8	−11.92	45.47	0.3
SJ (cm)	24.1 ± 1.7	25.3 ± 1.7	1.2	−3.02	0.62	0.2
CMJ (cm)	27.1 ± 1.9	27.1 ± 2.0	0.1	−1.49	1.35	0.0
**PERFORMANCE**						
Avg velocity 2 Km (km h^−1^)	13.9 ± 0.5	14.3 ± 0.5	0.4	−0.62	−0.18	0.1^*^

*Results are presented as mean ± SD*.

* *p value PRE vs. POST < 0.05*.

## Discussion

The first purpose of this study was to evaluate the effects of a different intensity distribution on laboratory tests and performance. Both groups, polarized and focused (intensity distribution 77/3/20 and 40/50/10, respectively) showed a significant improvement in velocity at V'O_2max_, VT, RCT, running economy and in performance on 2 km without a variation of value of V'O_2max_. There are no significant differences between groups that support one approach over another with recreational athletes. In the study of Muñoz et al. (2014) on recreational runners, they found improvement on 10 km performance between pre-post training, but no differences between groups that followed training program with emphasis on a polarized intensity distribution and threshold emphasis distribution. The changes recorded in our study are in agree with the results of several training studies reported using different training modalities for 8 weeks in recreational runners. In the recent study of Pugliese et al. (2018) there was an improvement in the speed at V'O_2max_ about +6% and speed at VT about +5% with any increment in V'O_2max_, while the improvement in performance on 5 km was about 3%. Similar results were observed also in master runners following concurrent strength and endurance training (Piacentini et al., 2013). Also the changes recorded for RE are aligned with changes reported in several study (Spurrs et al., 2003; Piacentini et al., 2013; Festa et al., 2019).

To date these are the only studies that have analyzed the effect of different intensity distribution on recreational runners. A study of Neal et al. (2013) observed superior performance effects of polarized training in a group of cyclist with a better fitness level to the current study runners. The study was well-controlled and the difference between the group was emphasized because they have eliminated all training above the RCT (zone 3) in their threshold group.

While there is strong agreement that polarized training model it is widely used among elite coaches and athletes, and several studies have shown that it allows them to achieve greater improvements in performance, on recreational athletes no evidence has yet emerged among the compared models.

This distribution is necessary for athletes who perform a large volume of training, to prevent overtraining or steady state of performance (Tarperi et al., 2017). Moreover, by accumulating less effort, the quality of high-intensity sessions is better and this could lead to a greater improvement compared to thresholds or focused model (Muñoz et al., 2014). Average volume for recreational runners is 3–5 h per week, and the possibility of overtraining occurring is very low, and they seem to show that they have a good tolerance to accumulate time at such intensity.

The limited volume of training hours for recreational athletes is determined primarily by the availability of time to train. The focused model seems to better meet the needs of recreational athletes to maximize the improvement from training.

The current study has some potential limitations regarding the lack of observed results on the effects of training on half marathon and marathon performances. However, in order to quantify any likely training effect, we performed a 2 km time-trials before and after the training intervention in both groups. Such shorter testing distance, even though not directly representative of a longer distance performance (e.g., half marathon), represents a more practical and time-effective way to quantify training effect. In future studies, it would be important to include some longer testing sessions to directly verify any training effect over performances more related to half and full marathon distance.

The results of this study seem to confirm the ability of recreational subjects to be equally sensitive to the different modes of intensity distribution over a limited period of time. Further studies regarding the long-term sustainability of this distribution method are required.

## Data Availability Statement

All datasets generated for this study are included in the article/supplementary material.

## Ethics Statement

The studies involving human participants were reviewed and approved by Ethical Committee for Experimentation of Department of Neuroscience, Biomedicine and Movement Science, University of Verona (Italy). The patients/participants provided their written informed consent to participate in this study.

## Author Contributions

CT and LF: conceptualization and formal analysis. CT, AL, and FS: methodology, supervision, and writing–review and editing. LF, CT, and KS: investigation. LF: data curation and writing–original draft preparation. FS: project administration.

### Conflict of Interest

The authors declare that the research was conducted in the absence of any commercial or financial relationships that could be construed as a potential conflict of interest.
